# Non-Invasive Characterization of Experimental Bone Metastasis in Obesity Using Multiparametric MRI and PET/CT

**DOI:** 10.3390/cancers14102482

**Published:** 2022-05-18

**Authors:** Gasper Gregoric, Anastasia Gaculenko, Lisa Nagel, Vanessa Popp, Simone Maschauer, Olaf Prante, Marc Saake, Georg Schett, Michael Uder, Stephan Ellmann, Aline Bozec, Tobias Bäuerle

**Affiliations:** 1 Institute of Radiology, Universitätsklinikum Erlangen, Friedrich-Alexander-Universität (FAU) Erlangen-Nürnberg, 91054 Erlangen, Germany; gasper.gregoric@fau.de (G.G.); lisa.nagel@uk-erlangen.de (L.N.); vanessa.popp@fau.de (V.P.); marc.saake@uk-erlangen.de (M.S.); michael.uder@uk-erlangen.de (M.U.); stephan.ellmann@uk-erlangen.de (S.E.); 2 Department of Internal Medicine 3, Rheumatology and Immunology, Universitätsklinikum Erlangen, Friedrich-Alexander-Universität (FAU) Erlangen-Nürnberg, 91054 Erlangen, Germany; anastasia.gaculenko@uk-erlangen.de (A.G.); georg.schett@uk-erlangen.de (G.S.); aline.bozec@uk-erlangen.de (A.B.); 3 Department of Nuclear Medicine, Universitätsklinikum Erlangen, Friedrich-Alexander-Universität (FAU) Erlangen-Nürnberg, 91054 Erlangen, Germany; simone.maschauer@uk-erlangen.de (S.M.); olaf.prante@uk-erlangen.de (O.P.)

**Keywords:** tumor-induced bone disease, adipocytes, bone μCT/MRI, functional imaging dce dwi pet, osteoimmunology, PPARγ

## Abstract

**Simple Summary:**

The bone marrow microenvironment, particularly adipocytes, plays a pivotal role as growth mediators of disseminated tumor cells in the bone marrow. In this prospective longitudinal study, we used multimodal and multiparametric imaging from magnetic resonance (MRI) and hybrid imaging (positron emission tomography and computed tomography; PET/CT) to non-invasively characterize the pathophysiologic processes of experimental bone metastases in obese and non-obese individuals. Obesity was induced by a high-fat diet (HFD) in rats and mice, resulting in enhanced glucose metabolism and angiogenic activity in metastatic bone lesions, as shown by significantly increased dynamic contrast-enhanced MRI and [^18^F]fluorodeoxyglucose PET/CT parameters in HFD-fed animals. These results were validated with immunohistochemistry and gene expression analysis. In conclusion, pathophysiological processes can be non-invasively assessed by MRI and PET/CT, opening novel avenues for quantitative assessment of follow-up and monitoring of treatment response in skeletal lesions.

**Abstract:**

The growth of primary tumors and metastases is associated with excess body fat. In bone metastasis formation, the bone marrow microenvironment, and particularly adipocytes, play a pivotal role as growth mediators of disseminated tumor cells in the bone marrow. The aim of the present study is to non-invasively characterize the pathophysiologic processes in experimental bone metastasis resulting from accelerated tumor progression within adipocyte-rich bone marrow using multimodal imaging from magnetic resonance imaging (MRI) and positron emission tomography/computed tomography (PET/CT). To achieve this, we have employed small animal models after the administration of MDA-MB 231 breast cancer and B16F10 melanoma cells into the bone of nude rats or C57BL/6 mice, respectively. After tumor cell inoculation, ultra-high field MRI and µPET/CT were used to assess functional and metabolic parameters in the bone marrow of control animals (normal diet, ND), following a high-fat diet (HFD), and/or treated with the peroxisome proliferator-activated receptor-gamma (PPARγ) antagonist bisphenol-A-diglycidylether (BADGE), respectively. In the bone marrow of nude rats, dynamic contrast-enhanced MRI (DCE-MRI) and diffusion-weighted imaging (DWI), as well as [^18^F]fluorodeoxyglucose-PET/CT([^18^F]FDG-PET/CT), was performed 10, 20, and 30 days after tumor cell inoculation, followed by immunohistochemistry. DCE-MRI parameters associated with blood volume, such as area under the curve (AUC), were significantly increased in bone metastases in the HFD group 30 days after tumor cell inoculation as compared to controls (*p* < 0.05), while the DWI parameter apparent diffusion coefficient (ADC) was not significantly different between the groups. [^18^F]FDG-PET/CT showed an enhanced glucose metabolism due to increased standardized uptake value (SUV) at day 30 after tumor cell inoculation in animals that received HFD (*p* < 0.05). BADGE treatment resulted in the inversion of quantitative DCE-MRI and [^18^F]FDG-PET/CT data, namely a significant decrease in AUC and SUV in HFD-fed animals as compared to ND-fed controls (*p* < 0.05). Finally, immunohistochemistry and qPCR confirmed the HFD-induced stimulation in vascularization and glucose activity in murine bone metastases. In conclusion, multimodal and multiparametric MRI and [^18^F]FDG-PET/CT were able to derive quantitative parameters in bone metastases, revealing an increase in vascularization and glucose metabolism following HFD. Thus, non-invasive imaging may serve as a biomarker for assessing the pathophysiology of bone metastasis in obesity, opening novel options for therapy and treatment monitoring by MRI and [^18^F]FDG-PET/CT.

## 1. Introduction

Obesity substantially contributes to cancer-related deaths, resulting in decreased survival rates for overweight patients with different primary tumors such as breast cancer [1,2,3,4]. Thereby, excess body fat has been associated with increased growth of primary tumors and metastasis in adipocyte-rich organs [5]. In particular, bone marrow is rich in adipocytes and, at the same time, a favorable environment for circulating tumor cells, as up to 80% of patients with advanced breast and prostate cancer present with metastasis into bone [6,7]. Patients with malignant melanoma show a mean survival of 3.6 months after bone metastasis diagnosis, and the incidence is positively correlated with obesity in a non-metastatic state [8,9].

Bone marrow, with its adipose tissue, is hormone-sensitive, endocrinologically active, and contributes to intra-osseous tumor growth by providing proliferating factors for tumor cells [10]. Adipocytes are metabolically active cells that induce the release of protumorigenic cytokines, such as CXCL1/2, IL6, TNF-alpha, and adipokines, resulting in bone metastasis formation and bone resorption [11]. Thus, activation of adipocytes in the bone marrow upregulates pathways associated with increased glycolysis and angiogenesis, resulting in proliferation, new vessel formation, and bone destruction in the skeleton [12].

Imaging bone metastases is highly challenging. According to the response evaluation criteria in solid tumors (RECIST) presented more than two decades ago, measurement of bone metastases was not possible [13]. In the updated RECIST 1.1, bone metastases are measurable if there is a soft tissue component that can be morphologically assessed [14]. In a recent review, novel options for the measurability of bone metastases were summarized, including functional and metabolic parameters from magnetic resonance imaging (MRI) and positron emission tomography (PET) [15]. Quantitative approaches for the non-invasive characterization of bone metastases are needed to (i) understand the pathophysiology of osseous lesions during bone colonization, (ii) enable early diagnosis of skeletal lesions, and (iii) assess treatment response in metastasis to bone [16,17].

Recently, functional and metabolic parameters in breast cancer bone metastases have been described with multimodal imaging, including dynamic contrast-enhanced MRI (DCE-MRI), diffusion-weighted imaging (DWI), and [^18^F]fluorodeoxyglucose-PET/CT ([^18^F]FDG-PET/CT) in an experimental study [18]. In obesity, the intraosseous processes during bone metastasis formation are currently not known on the functional and metabolic levels. Therefore, the aim of the present study is to assess the functional and metabolic parameters in experimental bone metastases of obese individuals using MRI and PET for quantification and characterization of processes such as glycolysis and angiogenesis.

## 2. Materials and Methods

### 2.1. Cell Lines (MDA-MB-231 and B16F10)

For the development of bone metastases in male rats, the human triple-negative breast cancer cell line MDA-MB-231 (American Type Culture Collection [ATCC]) was used. The cells were grown in Roswell Park Memorial Institute 1640 (RPMI-1640) Medium (Invitrogen, Carlsbad, CA, USA) enriched with 10% fetal calf serum (Sigma, Taufkirchen, Germany). Under controlled conditions (37 °C, 5% CO_2_, humidified atmosphere), the cells were passaged 2–3 times weekly. For the mouse metastasis experiments, B16F10 melanoma cells (ATCC) were used. The cells were cultured in Dulbecco’s Modified Eagle’s Medium (DMEM) (Gibco/life technologies, Carlsbad, CA, USA) supplemented with 10% FCS and 1% Penicillin/Streptomycin (Gibco/life technologies). Passaging of the cells was performed 2–3 times a week. For B16F10 inoculation, the cells were trypsinated with 0.25% Trypsin– ethylenediaminetetraacetic acid (EDTA) (Gibco/life technologies) at 37 °C for 10 min, counted, and adjusted for intratibial (i.t.) injection in phosphate-buffered saline (PBS) (Gibco/life technologies).

### 2.2. Animal Models of Bone Metastases (Rat and Mouse)

RNU strain male rats 4–6 weeks old were acquired from Charles River Laboratories (Sulzfeld, Germany) and were kept in a pathogen isolated environment in the local animal facility (PETZ–University of Erlangen-Nuremberg) for 5–7 weeks. Their general medical condition was carefully controlled, and they received unlimited access to nourishment and water. For the first part of the experiment, animals were separated into 2 groups of 25 animals and afterward fed with 2 distinguished pathogen-free diets, which differed in the proportion of fat. The first group received the normal diet–ND (Sniff # 1324, 10 kJ% fat; ssniff Spezialdiäten, Soest, Germany), the latter the high-fat diet–HFD (Open Source Diets, D12331: 59 kJ% fat with/sucrose Surwitdiet; Research Diets, Inc., New Brunswick, NJ, USA). After one week of acclimatization, anesthetized animals underwent surgery to induce tumor growth in the knee region of the right hind leg. After the preparation of vessels in the right inguinal area, the superficial epigastric artery was ligated, opened, and subsequently injected with 1.5 × 10^5^ MDA-MB-231 breast cancer cells. In this model of bone metastases, tumors start to grow in the distal femur and proximal tibia [17,19,20].

C57BL/6 male 6-week-old mice were purchased from Charles River Laboratories and kept in the local mouse facility for 1 week for adjustment. Afterward, the animals were fed either the ND or HFD for a total time of 8 weeks. After 6 weeks into the diet, B16F10 murine melanoma cells were applied at 1 × 10^4^ cells/50 µL per knee to the anesthetized animals intratibially (i.t.) according to a standard protocol [21]. The mice were monitored for 2 h after waking up from the surgery and were sacrificed 12 days post tumor inoculation for histology and qPCR analysis. The tumors mainly started to grow in the proximal metaphysis of the tibia, further extending into the distal tibia over time.

The study was not blinded; nonetheless, there existed no specific allocation procedure for assigning animals to corresponding groups. In the animal housing facility, animals were kept under controlled conditions (temperature, humidity, ≤5 animals/cage). At the end of the experiments, the animals were euthanized, and tissue samples were obtained for further examinations.

For the BADGE experiment, 18 RNU strain male rats were obtained and separated into 2 equal groups, which were fed with high fat or normal diet, respectively. In addition to the first experiment, after one week of acclimatization, i.p. BADGE (Sigma-Aldrich, St. Louis, MO, USA) injection occurred 3 times a week with a concentration of 120 mg/kg. BADGE application in mice was performed by i.p. injection of the compound at 30 mg/kg daily after tumor inoculation.

### 2.3. Image Acquisition (Rat)

RNU male rats were submitted to non-invasive imaging seven days post-diet starting. Subsequently, MDA-MB 231 was injected intraarterially (d0). Afterward, the imaging was conducted on days 10, 20, and 30 post-surgery. During the examination, animals were anesthetized using isoflurane (1.5% Isoflurane + 0.5 L/min). MRI imaging, including DCE and DWI, was performed on a 7-Tesla Ultra-High-Field MRI Scanner (ClinScan and BioSpec 70/30, Bruker, Ettlingen, Germany). Contrast agent gadobutrol (0.1 mmol/kg body weight) was slowly applied within approximately 10 s in the lateral tail vein of animals for the DCE imaging, 30 s after the start of the DCE sequence. PET/CT was performed on an Inveon preclinical hybrid scanner (Siemens Medical Solutions USA, Inc., Malvern, PA, USA). Preceding image acquisition, the radiotracer [^18^F]fluorodeoxyglucose ([^18^F]FDG) was intravenously injected with an activity of about 6 MBq. First, the CT images were obtained using the following CT parameters: duration 10 min, tube voltage 80 kV, tube current 500 µA, and isotropic resolution 48.9 µm. Immediately afterward, the PET imaging protocol was conducted (duration 15 min, lower/upper discriminatory level 350/650 keV, timing window 3.438 ns). The animals in all groups received scans with both imaging modalities (MRI and PET/CT) on the respective days. Regarding the order of imaging, MRI was performed first, and PET/CT afterward. 

### 2.4. Image and Statistical Analysis (Rat)

**Figure 1:**
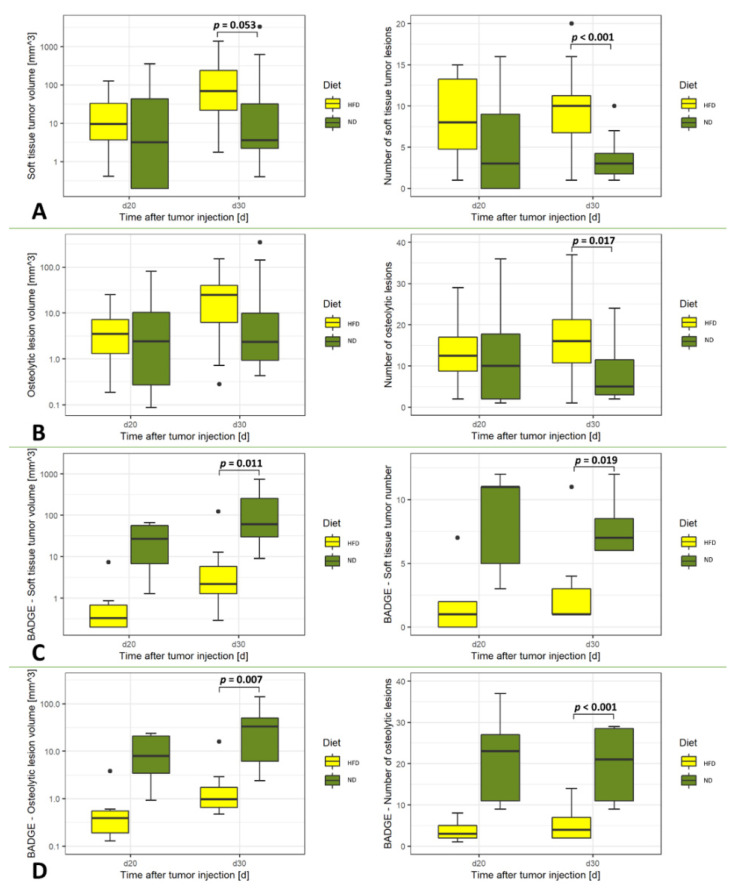
Morphologic tumor characteristics of the main (**A**,**B**) and the control (**C**,**D**) experiments. (**A**) Soft tissue tumor volume and number of lesions from MRI T2-weighted imaging (**B**) Osteolytic lesion volume and number of lesions determined with CT imaging. (**C**) Soft tissue tumor volume and number of lesions from MRI T2-weighted imaging under inhibition of the PPARγ pathway with BADGE (**D**) Osteolytic lesion volume and number of lesions determined with CT imaging under inhibition of the PPARγ pathway with BADGE. Volumes of osteolytic lesions and soft tissue tumors are given in mm^3^. Statistical analyses have been made with two-way ANOVA and Mann-Whitney-Wilcoxon U test. Significant values are defined as *p* < 0.05. Values of *p* are indicated up to a value of 0.15.

MRI and PET/CT images were analyzed using Horos open source DICOM viewer. Because the delineation of the tumor on DWI and DCE images tended to be difficult, the largest 2D extent of the tumor on T2 weighted MR images was circumscribed with a region of interest (ROI) and then copied and adjusted to DWI and DCE sequences. If the tumor was not macroscopically visible on days 10 and 20, the ROI with a size of 3 mm^2^ was placed in the bone marrow of the proximal tibia. The morphological information from MRI and CT (Figure 1A,B) overlaps with our previous report [20], which is presented here for comprehension. For the PET data, after fusion of CT and PET images, the oval ROI was placed on the coronar plane on the proximal tibia of the right hind leg, where due to the tumor metabolism, increased activity was perceived. Data for analysis were obtained directly from the ROI for the DWI and PET sequences. SUV value was calculated using the following equation:

(1) $$SUV=\frac{A_{ROI}\cdot m}{A_{inj}}$$

where $A_{ROI}$ represents the maximal activity of a chosen *ROI*, m is the animal weight and the $A_{inj}$ is the intravenously injected activity of a radioactive tracer. DCE tool plugin was used on the DCE images to obtain the time development of the signal after the contrast agent was injected, the so-called signal intensity versus time curve. Data were normalized using the average of the first 7 values, where no contrast agent was present. The data set was then analyzed with the parametric model of the signal curve, which yields parameters area under the curve (AUC), peak enhancement (PE), wash-in (WI), wash-out (WO), and time to peak (TTP).

Statistical computing and graphics were compiled with R programming language 4.1.12 using RStudio (RStudio PBC, Boston, MA, USA) as the integrated development environment. All independent variables of distinguished experimental groups were first tested with the Shapiro–Wilk test of normality. For the test values above 0.05, the normal distribution was assumed, and groups were compared with a statistical test two-way analysis of variance (two-way ANOVA). On the other hand, the Mann–Whitney–Wilcoxon U test was used for comparison of experimental groups when the Shapiro–Wilk test values were below 0.05, in which the data were assumed not to be normally distributed. *P*-values below *p* < 0.05 for both statistical tests, two-way ANOVA as well as Mann–Whitney–Wilcoxon U test, were considered statistically significant. On the figures, values of *p* are indicated up to a value of 0.15.

### 2.5. Immunofluorescence Staining (Rat and Mouse)

Immunofluorescence staining was performed in the rat (after inoculation of MDA-MB-231 breast cancer cells) and mouse (after inoculation of B16F10 melanoma cells) models.

After the final studies had been completed, the animals were sacrificed. For rats, the right hind legs were removed and prepared for fixation with 70% ethanol and embedded in Technovit^®^9100 NEW (Kulzer, Hanau, Germany). After removing the Technovit, the slides were treated with a target retrieval solution with a pH of 9 (Agilent Dako, Santa Clara, CA, USA). For immunofluorescence staining, Ki-67, Perilipin, and LDHA antibodies were used. In addition, the dye DAPI was used to counterstain the cell nuclei. 

After sacrificing the mice, tibiae were extracted, and surrounding soft tissue was removed. The bones were then preserved for 16 h in 4% paraformaldehyde (PFA) (Carl Roth, Karlsruhe, Germany) and washed twice with 70% ethanol (Carl Roth). For decalcification, the bones were placed into TEITEL buffer (14% EDTA-free acid, 9% NH_4_OH, pH 7.2) (VWR, Radnor, PA, USA) for 14 days at constant shaking. Afterward, the bones were washed twice with 70% ethanol (Carl Roth) and embedded in paraffin. The sections were cut at a thickness of 1 µm and placed on glass slides. For immunofluorescence staining, the slides were deparaffinized using a standard melting/rehydrating sequence: melting at 65 °C for 1 h, 3 × xylene for 5 min, 2 × 100% 2-propanol for 5 min, 2 × 95% 2-propanol for 5 min, 2 × 70% 2-propanol for 5 min, 1 × phosphate-buffered saline (PBS) for 5 min (Carl Roth). Subsequently, epitope retrieval was performed using tris(hydroxymethyl)aminomethane-EDTA buffer (Tris-EDTA buffer) (VWR) at 90 °C for 30 min. The blocking procedure was performed by applying 5% bovine serum albumin (BSA) for 1 h at RT. Further, the slides were washed twice with 1×PBS, and the respective first antibody was applied in 5% BSA (1:50 Ki67, Biozol, Eching, Germany or 1:50 CD31, Biolegend, San Diego, CA, USA). The slides were then incubated for 16 h at 4 °C before washing them again twice with PBS and applying the secondary antibody (1:150, Biolegend) in 5% BSA. Finally, after two additional washing steps with PBS, the slides were covered with Vectashield mounting medium with DAPI (Vector Laboratories, Burlingame, CA, USA). The slides were then mounted with a thin glass cover and further processed with a BZ-X710 all-in-one fluorescence microscope (Keyence Corporation of America, Itasca, IL, USA).

### 2.6. Gene Expression Analysis (Mouse)

Mouse bone marrow was flushed from the bone using a standard technique [22], resuspended in RNA-Solv (VWR), frozen, and stored at −80 °C until use. After thawing the bone marrow samples on ice, they were processed according to the manufacturer’s manual, using chloroform (Carl Roth), 100% 2-propanol, and 70% ethanol. The RNA pellets were resuspended in nuclease-free water (Qiagen, Venlo, Netherlands) and subsequently analyzed for quantity/quality with a Nanodrop 2000 (Thermo Fisher Scientific, Waltham, MA, USA). For cDNA synthesis, a high-capacity cDNA reverse transcription kit (Life Technologies, Inc., Grand Island, NY, USA) was used. Afterward, gene expression analysis was performed using quantitative real-time polymerase chain reaction (qPCR) with an SYBR select master mix (Applied Biosystems, Foster City, CA, USA) using a Quantstudio 6 Flex and its software (Applied Biosystems). The primers used are listed in Table S1.

## 3. Results

For our study, a total of 65 rats were inoculated with MDA-MB 231 tumor cells. Seven days before tumor cell inoculation, rats received either ND or HFD. After initial imaging on day 0, rats were divided into 4 groups and 2 experiments. In the main experiment (*n* = 47 rats), 24 rats received high fat and 23 rats normal diet. The rats were followed-up by imaging on days 10, 20, and 30 after tumor cell inoculation. In the control experiment, all animals (*n* = 18 rats) were injected with BADGE, while 9 rats received high fat and normal diet, respectively.

For validation purposes of non-invasive imaging, immunohistology staining for factors of vascularization (only in mice), proliferation, or glycolysis (only in rats) was performed from bone metastases in rats (after inoculation of MDA-MB 231 tumor cells) and mice (after inoculation of B16F10 melanoma cells). Finally, a gene expression analysis by qPCR quantified factors from vascularization and glycolysis derived from murine skeletal lesions (after inoculation of B16F10 melanoma cells).

### 3.1. Main Experiment

Of the 24 rats in the high-fat diet group, 16 developed osteolytic bone metastases in the hind leg (67%), while only 12 out of 23 rats (52%) were diagnosed with a bone lesion in the observation period. The body weight of the animals increased in both groups from days 10 to 30 after tumor cell inoculation, with significantly heavier rats at day 30 in the high-fat diet group (Figure S1).

The results from morphological imaging by computed tomography and magnetic resonance imaging showed that the number of the osteolytic lesions (CT) and the soft tissue tumors (MRI) were higher in animals in the HFD group at day 30 as compared to those fed with the ND, respectively (*p* < 0.001, soft tissue tumors and *p* = 0.017, osteolytic lesions, Figure 1).

**Figure 2:**
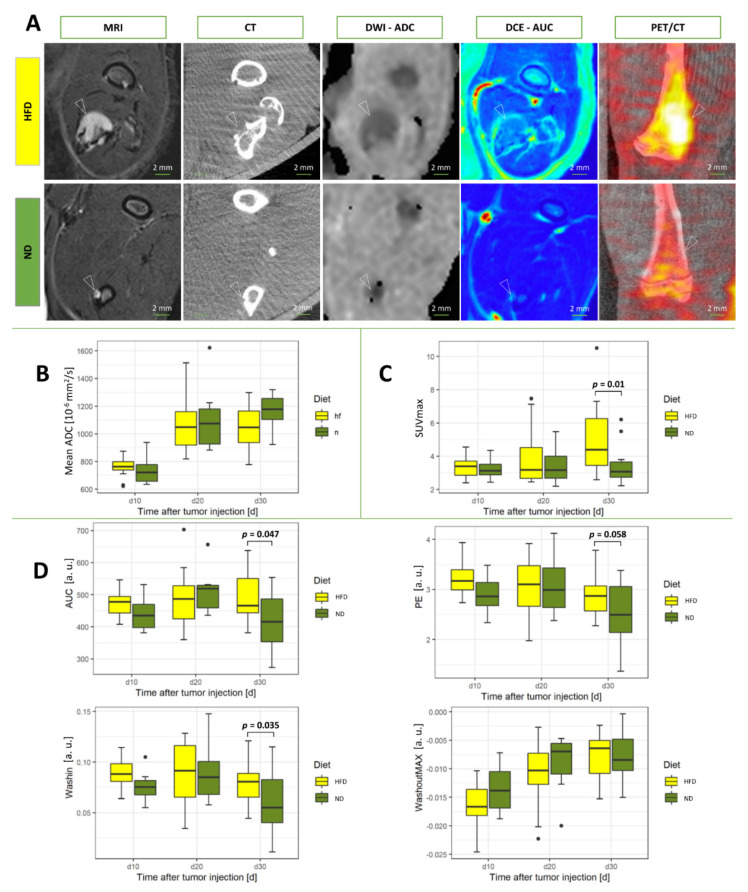
Functional and metabolic parameters of the main experiment from MRI and [^18^F]FDG−PET/CT. (**A**) Representative images for day 30 of multiparametric MRI and PET/CT for both diet regimes. (**B**) Apparent diffusion coefficient (ADC) from diffusion-weighted imaging (**C**) Standardized uptake value (SUV) from positron emission tomography (PET). (**D**) Calculated parameters for the quantitative model of the signal intensity versus time curve of the dynamic contrast-enhanced (DCE) MR perfusion. White arrows indicate location of the bone metastases. Abbreviation a.u. stands for arbitrary units. Statistical analyses were performed with two-way ANOVA and Mann-Whitney-Wilcoxon U test. Significant values are defined as *p* < 0.05. Values of *p* are indicated up to a value of 0.15.

Metabolic imaging from [^18^F]FDG-PET/CT revealed significantly increased SUVmax in rats from the HFD group (*p* = 0.01; Figure 2). On the contrary, diffusion-weighted imaging from MRI did not show significantly different apparent diffusion coefficients (ADC) between the groups (Figure 1).

Functional imaging derived from dynamic contrast-enhanced magnetic resonance imaging displayed significant differences between the groups at day 30 for the parameters area under the curve (AUC; *p* = 0.047) and wash-in (WI; *p* = 0.035; Figure 2). Thereby, significantly increased parameters were assessed in the HFD group as compared to rats fed with the ND. The parameters peak enhancement (PE) and wash-out (WO) were higher in the HFD group but did not reach significance (PE, *p* = 0.058; WO, *p* = 0.321).

### 3.2. Control Experiment

In animals in the HFD and ND groups injected with BADGE, the number of rats that developed osteolytic bone lesions did not differ between the groups, with 7 out of 9 rats (78%) diagnosed with osseous metastases in both groups (Figure 1). Also, the weight of the rats from both groups did not differ significantly after administration with HFD or ND at days 10, 20, and 30 after tumor cell inoculation (Figure S1).

When comparing the number and volumes of the osteolytic lesions (CT) and soft tissue components of bone metastases (MRI), significantly smaller tumors were assessed by imaging in ND rats as compared to animals with HFD at day 30 (soft tissue tumor volume *p* = 0.011, soft tissue tumor number *p* = 0.019, osteolytic lesion volume *p* = 0.007 and number of osteolytic lesions *p* < 0.001, Figure 1).

**Figure 3:**
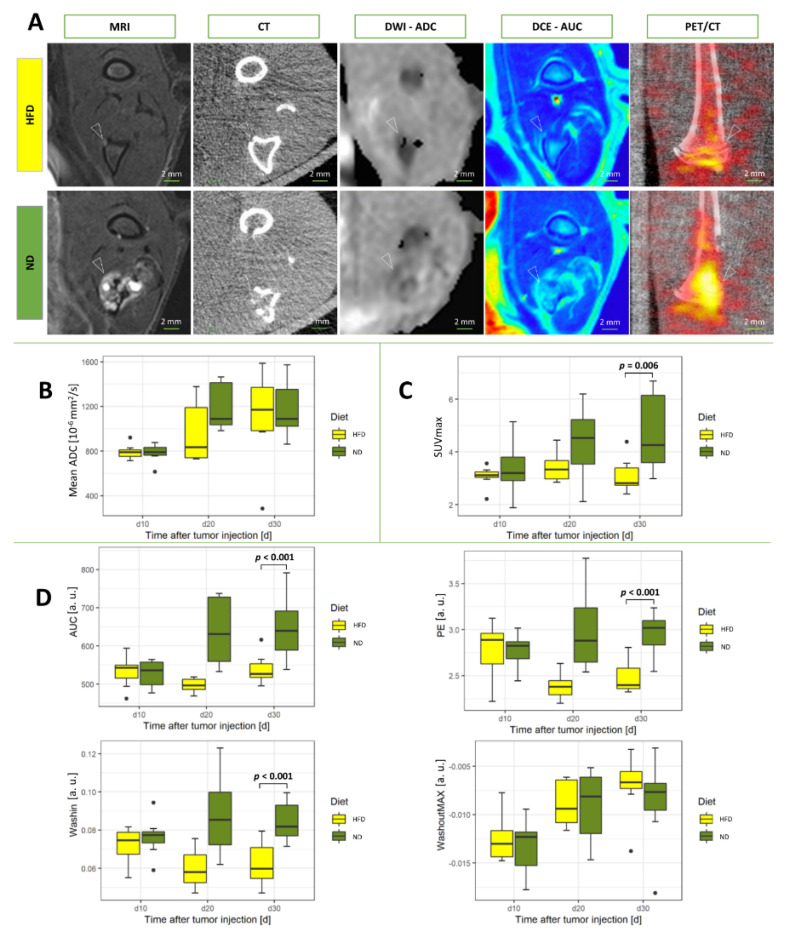
Functional and metabolic parameters of the control experiment in high fat and normal diet rats under inhibition of the PPARγ pathway with BADGE: (**A**) Representative images for day 30 of multiparametric MRI and PET/CT for both diet regimes under BADGE treatment (**B**) Apparent diffusion coefficient (ADC) of the diffusion-weighted imaging. (**C**) Standardized uptake value (SUV) of the positron emission tomography (PET). (**D**) Calculated parameters for the quantitative model of the signal curve of the dynamic contrast-enhanced (DCE) MR perfusion for BADGE experiment. White arrows indicate location of the bone metastases. Abbreviation a.u. stands for arbitrary units. Statistical analyses were performed with two-way ANOVA and Mann-Whitney-Wilcoxon U test. Significant values are defined as *p* < 0.05. Values of *p* are indicated up to a value of 0.15.

In contrast to the main experiment, SUVmax from [^18^F]FDG-PET/CT was significantly smaller in bone metastases from HFD rats as compared to rats receiving ND (*p* = 0.006; Figure 3). The apparent diffusion coefficient (ADC) from diffusion-weighted imaging did not differ significantly between the groups (*p* = 0.33; Figure 3).

Functional MR imaging using intravenous contrast (DCE-MRI) resulted in significantly decreased parameters area under the curve (AUC), wash-in (WI), and peak enhancement (PE) in rats from the HFD group (all *p*-values *p* < 0.001, respectively), while the parameter wash-out (WO) was not significantly different between animals of the HFD and ND groups (*p* = 0.34; Figure 2).

### 3.3. Immunohistochemistry

**Figure 4:**
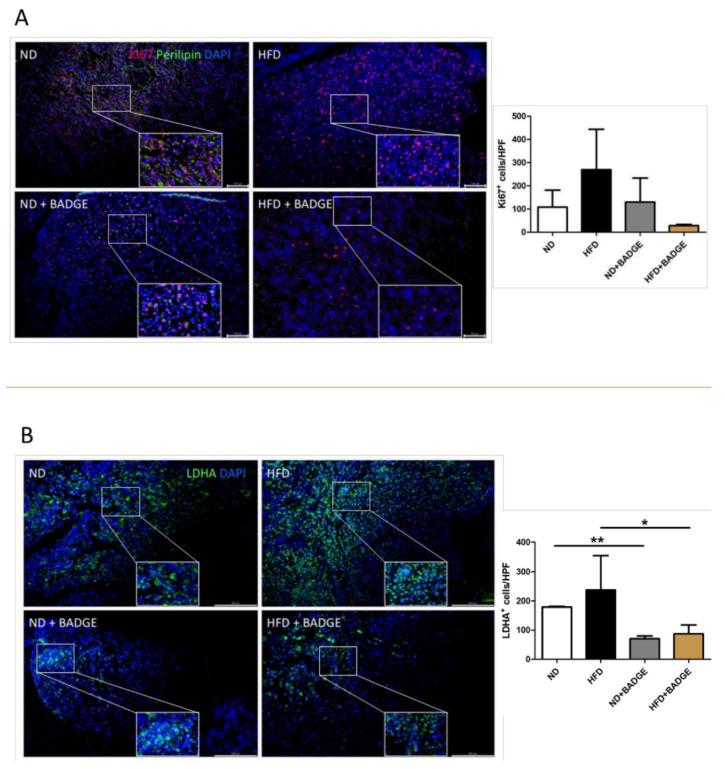
Immunofluorescence staining (rat) for proliferation and glycolysis marker in bone metastases after inoculation of MDA-MB 231 breast cancer cells. (**A**) Bone slides of ND, HFD, ND + BADGE, and HFD + BADGE animals were stained for the proliferation marker Ki-67 (red) and the adipocyte marker Perilipin (green). Cell nuclei were counterstained with DAPI (blue). Statistical analysis of Ki-67^+^ cells/HPF is shown (mean and standard deviation). (**B**) Bone slides of ND, HFD, ND + BADGE, and HFD + BADGE animals were stained for the glycolysis marker LDHA (green). Cell nuclei were counterstained with DAPI (blue). Statistical analysis of LDHA^+^ cells/HPF is shown (mean and standard deviation). * *p* < 0,05; ** *p* < 0,01. Scale bar = 100 μm.

Bone metastasis slides from rats inoculated with MDA-MB 231 breast cancer cells were stained for the makers of proliferation (Ki-67) and glycolysis (LDHA). Analysis of positive cells per high power field (HPF) revealed more proliferative and glycolytic activity after HFD (*p* > 0.05; Figure 4). After treatment with BADGE, decreased numbers of LDHA-positive cells were recorded as compared to the groups of the main experiments receiving ND and HFD only (*p* = 0.004, ND + BADGE; *p* = 0.04, HFD + BADGE). BADGE treatment reduced the number of Ki67-positive cells but did not reach significance (*p* > 0.05, ND + BADGE and HFD + BADGE; Figure 4).

**Figure 5:**
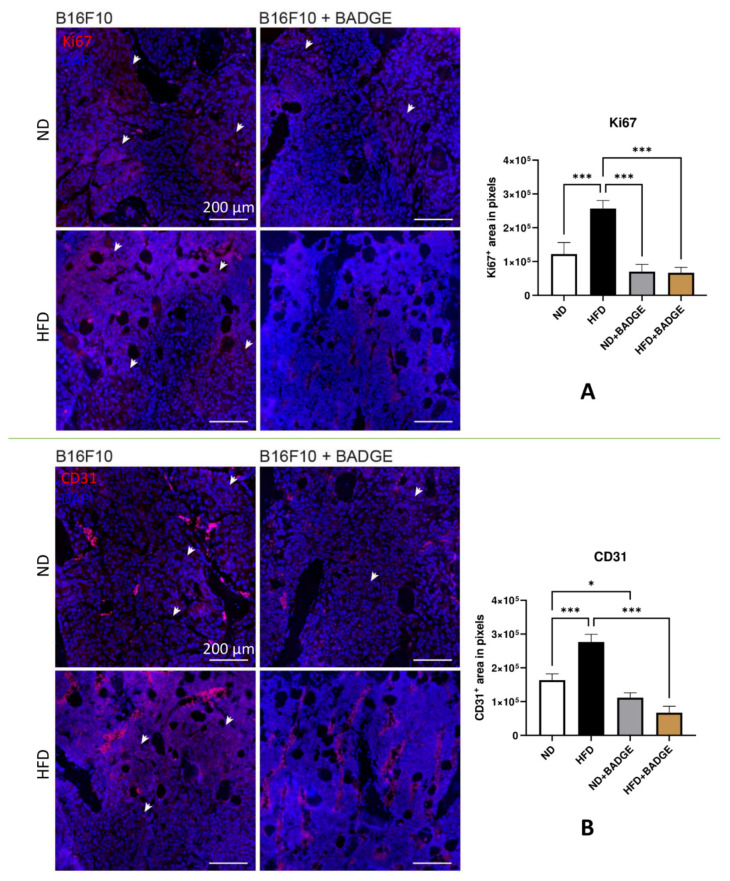
Immunofluorescence staining (mouse) for proliferation and vascularization markers in bone metastases after inoculation of B16F10 melanoma cells. (**A**) Bone slides of ND, HFD, ND + BADGE, and HFD + BADGE animals with tumors were stained for the proliferation marker Ki-67 (red). Cell nuclei were counterstained with DAPI (blue). (**B**) Bone slides of ND, HFD, ND + BADGE, and HFD + BADGE animals with tumors were stained for the vascularization marker CD31 (red). Cell nuclei were counterstained with DAPI (blue). White arrows indicate either Ki67- (top) or CD31 (bottom) positive areas. * *p* < 0,05; *** *p* < 0,001. Scale bar = 200 μm.

Bone metastasis slides from mice inoculated with B16F10 melanoma cells were stained for proliferation marker (Ki-67) and vascularization (CD31). Thereby, BADGE treatment decreased Ki67 proliferation and CD31-vascularization signal predominantly in HFD-fed mice. Representative images of immunofluorescence staining of paraffin-embedded mouse bones under different conditions are shown in Figure 5.

Bone metastasis slides from mice inoculated with B16F10 melanoma cells were stained for markers of proliferation (Ki-67) or vascularization (CD31). Analysis of the slides revealed the positively stained area. Here, BADGE treatment decreased Ki67 proliferation (*p* > 0.05, ND + BADGE; *p* < 0.001, HFD + BADGE) and CD31-vascularization (*p* = 0.041, ND + BADGE; *p* < 0.001, HFD + BADGE) signal predominantly in HFD-fed mice. Representative images of immunofluorescence staining of paraffin-embedded mouse bones under different conditions are shown in Figure 5.

### 3.4. Gene Expression Analysis

**Figure 6:**
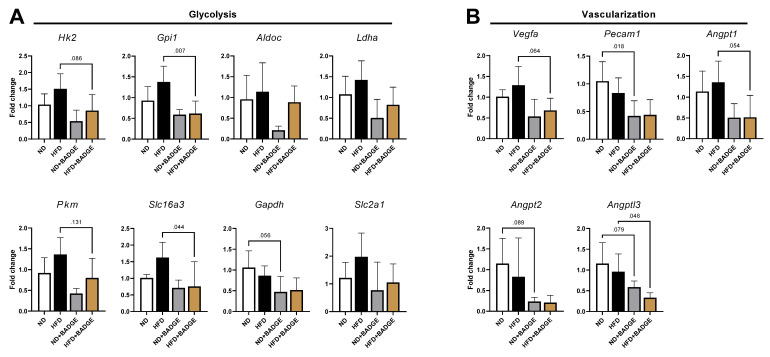
Antagonism of PPARγ in tumor-bearing mice decreased glycolytic and vascularization markers. (**A**) Gene expression markers for glycolysis. (**B**) Gene expression markers for vascularization. Relative gene expression in normally fed tumor-bearing mouse bone marrow normalized to ND with tumors. Values of *p* indicated up to a value of 0.15 (only decimal places given).

Mouse B16F10-induced tibial metastases were analyzed 12 days post tumor cell inoculation for gene expression by extracting the bone marrow. When comparing ND to HFD, there was a non-significant tendency to increase expression of factors associated with glycolysis and vascularization in HFD as compared to ND (Figure 6). When BADGE treatment was applied for both diets, glycolytic and angiogenic markers were down-regulated, reaching significance for HFD in the case of glucose-6-phosphate isomerase 1 (Gpi1, *p* < 0.05), solute carrier family 16 member 3 (Slc16a3, *p* < 0.05), and angiopoietin-like 3 (Angptl3, *p* < 0.05). For ND mice treated with BADGE, only platelet/endothelial cell adhesion molecule 1 showed significance (Pecam1, *p* < 0.05). The primers used for the expression analysis are summarized in Table S1.

## 4. Discussion

Obesity is a major factor influencing the growth of metastatic lesions. Here, we used multimodal and multiparametric imaging from MRI and PET/CT to characterize the intraosseous processes of experimental bone metastases in a longitudinal study producing novel non-invasive biomarkers. In the presented experimental model, obesity was induced by HFD in rats and mice, resulting in enhanced glucose metabolism and angiogenic activity in metastatic bone lesions as shown by significantly increased DCE-MRI and [^18^F]FDG-PET/CT parameters in HFD-fed animals, and validated with immunohistochemistry and gene expression analysis. 

Most cancer cells employ oxygen-independent glycolysis even in the presence of oxygen, which is referred to as the Warburg effect. Along with other researchers, we previously demonstrated that bone marrow adiposity might fuel tumor cells with fatty acids, interleukins, and cytokines, e.g., osteopontin [23,24], most probably resulting in the enhanced glycolytic activity of tumor cells, which is then captured by [^18^F]fluorodeoxyglucose metabolism and increased SUVmax values, as determined in the present study. 

MDA-MB 231 breast cancer and B16F10 melanoma cells used in the present study highly express enzymes of the glycolytic pathway, such as phosphoglycerate kinase and proteins associated with the pentose phosphate pathway [25]. Moreover, these osteotropic cells produce large amounts of lactate, which is released by monocarboxylate transporter 4 and taken up by osteoclasts through the transporter MCT1, fueling their oxidative metabolism and promoting osteoclast-mediated bone resorption [12,26]. As a consequence of the administration of the adipocyte differentiation inhibiting agent, BADGE, a significant decrease in SUVmax could be recorded in HFD rats compared to those receiving a regular diet. The parameter SUVmax from [^18^F]FDG-PET/CT in bone metastases can be therefore considered as an adequate biomarker for adipocyte-induced glycolysis in breast cancer bone metastases.

Another finding from the non-invasive imaging in the present study was the significant increase in DCE-MRI parameters such as AUC and PE in HFD-fed rats and a significant decrease in obese animals receiving BADGE treatment, as compared to regularly fed control rats, respectively. In line with previous reports, angiogenesis is increased in bone metastasis formation by activation of pro-angiogenic factors such as VEGF, VEGFR, placental growth factor, and integrins [27]. In particular, DCE-MRI was capable of diagnosing a decrease in angiogenic activity in experimental bone metastases by semiquantitative measures or pharmacokinetic modeling [28]. Due to inhibition of crucial angiogenic factors VEGF, VEGFR, and integrins alpha v beta 3/5 by tyrosine kinase inhibitors (sunitinib, sorafenib), neutralizing antibodies (avastin), or small molecule inhibitors (cilengitide), significant alterations in DCE-MRI parameters could be quantitatively determined in bone metastases [29,30,31,32]. As angiogenesis was also found to be upregulated in metabolically active adipose tissue in bone [33,34], DCE-MRI parameters present another promising biomarker to assess the progression of bone metastases in adipocyte-rich bone marrow.

Assessment of treatment response is a major challenge in bone metastasis. The standard procedure for tumor response assessment in bone metastasis is RECIST 1.1 (response evaluation criteria in solid tumors). In this criteria, only bone metastases with a discernible soft tissue mass are measurable, hampering the clinical usability of this technique [14]. Thus, quantitative techniques capturing pathophysiologic information about bone metastases are a major chance to improve treatment monitoring in bone metastases [15]. 

In our present study, [^18^F]FDG-PET/CT and DCE-MRI parameters indicated treatment response following BADGE administration resulting in significant differences between animals fed with HFD and ND, respectively. Therefore, these multiparametric and multimodal parameters are promising for adequate quantification of treatment response in bone metastases. As a major advantage, the combination of these techniques enables assessing osteolysis (CT), soft tissue metastases (MRI), vascularization (DCE-MRI), diffusion (DWI), and glucose metabolism ([^18^F]FDG-PET/CT), which can all be determined quantitatively. Consequently, all morphologically and functionally relevant changes that are influenced by current therapeutic approaches can be captured [15].

Nevertheless, our study has limitations, including the number of animals recruited for this study and the applied diet regimen administered to the animals. The number of animals was limited to at least 23 rats per group for the main experiment and 9 animals per group for the control experiment, respectively, without performing statistical tests for power requirements. However, due to the longitudinal character of the study, we acquired three follow-up scans per animal and thereby increased the number of available datasets by three times. Secondly, for an optimized tumor take rate, we started with the specific diet only seven days prior to tumor cell inoculation. Indeed, a longer time period for the diet might have enhanced bone marrow adiposity, but from earlier experiments, we have learned that the tumor take rate decreases with the increasing age of animals [19]. Thirdly, as this is a preclinical study, the clinical benefit of the presented results is currently unclear. Here, obesity was induced by a high-fat diet over several weeks resulting in a relatively fast increase in body fat, including bone adiposity, which is not paralleled by clinical situations. In the clinical situation, multiple factors and a longer period of time result in obesity. However, the imaging techniques used in this study for MRI and PET/CT were performed according to the clinical regime, which should be comparable regarding image assessment and post-processing, including quantification applied for diagnostics of bone metastases in breast cancer and melanoma patients.

## 5. Conclusions

In this prospective longitudinal study, we present quantitative biomarkers for perfusion and metabolism of experimental bone metastases in obese individuals. Thereby, pathophysiological processes can be non-invasively assessed by MRI and PET/CT, opening novel avenues for quantitative assessment of follow-up and monitoring of treatment response in skeletal lesions.

## Data Availability

All data acquired for this study are stored at the data center of the University Hospital Erlangen, Institute of Radiology.

## Supplementary Materials

The following supporting information can be downloaded at: https://www.mdpi.com/article/10.3390/cancers14102482/s1, Figure S1: Macroscopic features of the experiment with bone metastasis development in HFD and ND rats. (A) Tumor development in rats after MDA-MD-231 cell inoculation—Tumor take rate. (B) Weight comparisons between HFD and ND in main and control experiment. Statistical analyses have been made with two-way ANOVA. Significant values are defined as *p* < 0.05. Values of *p* are indicated up to a value of 0.15; Table S1: qPCR primers used for analysis of glycolytic and angiogenic genes.
