# Supplementary material for: Non-Invasive Characterization of Experimental Bone Metastasis in Obesity Using Multiparametric MRI and PET/CT
Source: Cancers (Basel). 2022 May 18;14(10):2482. doi: 10.3390/cancers14102482 (PMC9139574; doi:10.3390/cancers14102482)
Supplement: Supplementary file 1 [file cancers-14-02482-s001.zip › cancers-1696704-supplementary.pdf]

Supplementary Materials: Non-Invasive Characterization of Experimental Bone Metastasis in Obesity Using Multiparametric MRI and PET/CT

Tumor take rate

**HFD**	67 % (16/24)
**ND**	52 % (12/23)

Tumor take rate - Badge

**HFD**	78 % (7/9)
**ND**	78 % (7/9)

A

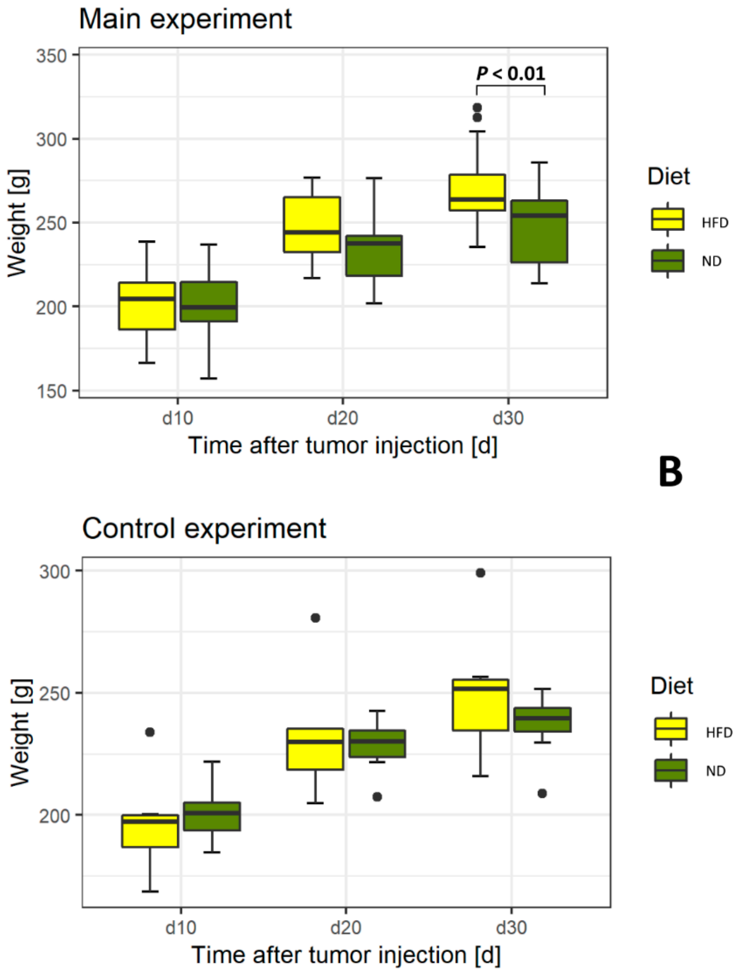

Figure S1. Macroscopic features of the experiment with bone metastasis development in HFD and ND rats. (**A**) Tumor development in rats after MDA-MD-231 cell inoculation—Tumor take rate. (**B**) Weight comparisons between HFD and ND in main and control experiments. Statistical analyses have been made with two-way ANOVA. Significant values are defined as $p < 0.05$. Values of p are indicated up to a value of 0.15.

Table S1. qPCR primers used for analysis of glycolytic and angiogenic genes.

Target	Enzymes	Forward sequence	Reverse sequence
		Housekeeping	
Actb	beta actin	TGTCCACCTTCCAGCAGATGT	AGCTCAGTAACAGTCCGCCTAGA
		Glycolysis	
Hk2	hexokinase 2	GGAGAGCACGTGTGACGAC	GATGCGACAGGCCACAGCA
Gpi1	glucose-6-phosphate isomerase 1	GTTGCCTGAAGAGGCCAGG	GCTGTTGCTTGATGAAGCTGATC
Aldoc	aldolase C, fructose-bisphosphate	GGCAGAGATGAACGGGCTTG	GGCGATGTAGAGGGACTGTG
Ldha	lactate dehydrogenase A	CCAGCAAAGACTACTGTGTAACTG	AGATGTTCACGTTTCGCTGGA
Pkm	pyruvate kinase	CAGGAGTGCTCACCAAGTGG	CATCAAGGTACAGGCACTACAC
Slc16a3	solute carrier family 16 member 3	TGGCATCTCATATGGCATGGTG	CACCTCCTCAGGCTCTGTC
Slc2a1	solute carrier family 2 member 1	TTAATCGCTTTGGCAGGCGG	CCCAGTTTGGAGAAGCCCAT
Gapdh	glyceraldehyde-3-phosphate dehydrogenase	CATCACTGCCACCCAGAAGACTG	ATGCCAGTGAGCTTCCCGTTCAG
		Vascularization	
Vegfa	vascular endothelial growth factor A	CTGCTGTAACGATGAAGCCCTG	GCTGTAGGAAGCTCATCTCTCC
Pecam1	platelet/endothelial cell adhesion molecule 1	CCAAAGCCAGTAGCATCATGGTC	GGATGGTGAAGTTGGCTACAGG
Angpt1	angiopoietin 1	CCAGGCCCGTTGTTCTTGAT	GGAAGGGAGACTTGCTCATTC
Ang2	angiogenin member 2	AGAATAAGCAAGTCTCGCTTCC	TGAACCCTTTAGAGGCTCGGT
Angptl3	angiopoietin-like 3	TGATACCCAATCAGGCAGTCC	GACTGCCCAGGTGAAAGGAG
